# Epidemiology, length of stay, and survival outcomes of *Candida auris* infections in a tertiary care center in the greater detroit area

**DOI:** 10.1017/ash.2025.10237

**Published:** 2025-11-27

**Authors:** Hussein Tehaili, Shayan Sharifi, Sruti Ramamoorthy, Melike Yildirim, Prabhnoor Dhillon, Raveen Mahngar, Lavina Jabbo, Marco R. Scipione, Hossein Salimnia, Glenn Tillotson, Teena Chopra

**Affiliations:** 1 School of Medicine, Division of Infectious Diseases, Wayne State University, Detroit, MI, USA; 2 Department of Industrial and Systems Engineering, Wayne State University, Detroit, MI, USA; 3 School of Medicine, Wayne State University, Detroit, MI, USA; 4 Family Medicine and Public Health Sciences, Wayne State University, Detroit, MI, USA; 5 Adult Central Campus Infection Control and Epidemiology Department, Detroit Medical Center, Detroit, MI, USA; 6 Detroit Receiving Hospital, Detroit, MI, USA; 7 Detroit Medical Center, Microbiology Division, Detroit, MI, USA; 8 School of Medicine, Department of Pathology, Wayne State University, Detroit, MI, USA; 9 GST Micro North, VA, USA

## Abstract

**Objectives:**

To investigate the epidemiology and clinical outcomes of *Candida auris* (*C. auris*), reclassified *Candidozyma auris*, in a Detroit tertiary care center, focusing on mortality, length of stay (LOS), glucose variability, and demographic factors.

**Setting:**

Inpatient admissions in a Detroit tertiary care center, June 2023–March 2025.

**Patients:**

Among 9,025 *C. auris* tests, 725 (8.03%) were positive. A subpopulation of 242 hospitalized patients was analyzed for outcomes.

**Methods:**

Clinical and demographic data, including infection status, mortality, and LOS, were analyzed. Glucose variability was assessed by the coefficient of variation. ANOVA and Kruskal–Wallis tests evaluated group differences, pairwise Z-tests compared mortality, and a competing-risks survival analysis estimated probabilities of discharge and in-hospital death.

**Results:**

Mortality was highest among colonized/infected patients (31.3%) and significantly associated with race (*p* = 0.0072) and ethnicity (*p* = 0.0069). High glucose variability (>190 mg/dL) was linked to increased mortality (27.63%) and prolonged LOS (46.14 days; *p* < 0.01). Infected patients had the longest LOS (67.9 days), followed by colonized/infected (39.9) and colonized-only (30.2; *p* < 0.001).

**Conclusion:**

*C. auris* disproportionately affects African American patients and those with high glucose variability, contributing to higher mortality and longer hospitalization. Infection status and glycemic instability were the strongest predictors of LOS, while racial and ethnic disparities influenced mortality. Strengthened infection control, early identification, and optimized glucose management may improve outcomes in high-risk populations.

## Introduction

*Candida auris*, recently reclassified as *Candidozyma auris* (hereafter *C. auris*), is an emerging multidrug-resistant pathogen that poses a growing public health threat in healthcare settings. First identified in Japan in 2009, it has since spread globally, with the first U.S. cases reported in 2016.^1^ Its persistence on surfaces facilitates transmission and complicates outbreak control.^1–3^

*C. auris* hospitalizations rose sharply between 2017 and 2022, reflecting increasing prevalence and containment challenges.^3,5^ Resistance to standard disinfection methods hinders infection control, especially in long-term and intensive care settings.^5^ Prolonged hospitalizations are associated with higher colonization and infection rates, reflecting patient complexity. ^3,10^ This national burden is mirrored in Detroit, where outbreaks are increasingly reported.^6^ Many occur in high-acuity or long-term facilities, where delayed identification and limited infection control exacerbate spread.^7^ Asymptomatic colonization contributes to ongoing transmission of *C. auris* in Michigan.^2,8^ Detroit’s healthcare system serves a predominantly African American population with high chronic disease prevalence, increasing infection risk.^9^ Prior studies indicate higher candidemia rates in this group, a disparity that extends to *C. auris*.^10^

The present study investigates the epidemiology and outcomes of *C. auris* in a tertiary care center in urban Detroit. This study examines key clinical parameters, including mortality, length of stay (LOS), and blood glucose variability, among hospitalized patients with *C. auris*. Given the association between hyperglycemia and infection severity, the role of glucose instability in *C. auris* outcomes is also explored.^11^

## Methods

### Study design and data collection

This retrospective cohort study evaluated clinical outcomes of patients with *C*. *auris* infections. Evaluations included: (1) all *C. auris* cases (colonized and infected patients) for broader epidemiological insights, and (2) a subpopulation analysis to gain detailed clinical outcomes.

Data were collected from five Detroit hospitals within the same tertiary center for admissions between June 2023 and March 2025. Culture followed by MALDI-ToF and/or an in-house, real-time, qualitative PCR was used for detection/identification of *C. auris* in patient samples. A total of 9,025 *C. auris* PCR tests were performed, of which 725 were positive (8.03%). The subpopulation analysis included all 242 unique hospitalized patients with a first positive *C. auris* test result from June 2023 to February 2024. For prevalence and outcome analyses, only the first positive *C. auris* test per patient was included. Multiple samples from the same individual were not counted separately, ensuring that each patient contributed a single index event.

### Definitions and key variables

Patients were categorized as colonized, infected, or colonized/infected according to infection control protocols. Patients identified by PCR screening alone were classified as colonized. Patients with positive clinical cultures (eg, blood, urine, or other sites) were classified as infected. Those with sequential PCR screening and subsequent positive clinical cultures were classified as colonized/infected. Classification was based solely on laboratory detection and not on provider diagnosis, documentation, or treatment response. Because culture positivity from nonsterile sites such as urine or sputum may reflect colonization rather than true infection, this distinction is noted as a limitation.

Age at admission during index hospitalization (during which *C. auris* was first detected) was recorded continuously and grouped into four categories (18–40, 41–60, 61–80, 80+ years) to evaluate age-specific outcomes, including LOS and mortality. Similarly, sex was recorded as a binary variable (male or female). Ethnicity was categorized into Not Hispanic or Latino, Hispanic or Latino, Multiple Ethnicities, or Unknown; race as African American, White, Multiple, and Other. LOS was measured as time from hospital admission to discharge or in-hospital death.

Glucose levels were analyzed as a continuous variable, while glucose variability was defined as the difference between the maximum and minimum glucose levels recorded for each patient during hospitalization. This operational definition was selected to allow standardized extraction across patients with variable testing frequency. Although other metrics (eg, lability index or SD) may offer more granularity, they were not consistently available in our data set. Based on this, patients were categorized into three groups: low variability (<84 mg/dL), medium variability (84–190 mg/dL), and high variability (>190 mg/dL).

Glucose stability, measured using the coefficient of variation (CV), was also categorized into three groups: high stability (low CV), medium stability, and low stability (high CV). The CV, a widely used metric in variability analysis, was calculated from multiple glucose readings over a specified period, offering a robust measure of fluctuations in glucose levels. Patients were grouped into low CV (<20%), moderate CV (20–36%), and high CV (>36%). A low CV indicated more consistent glucose levels across repeated measurements.

This categorization enabled us to examine the relationship between glucose and key outcomes, including LOS, defined as the total number of days hospitalized from admission to discharge, and mortality, assessed as a binary outcome (alive or deceased).

### Statistical approaches

Differences in LOS and mortality across subgroups were assessed using parametric and non-parametric methods. The Shapiro-Wilk test was used to assess normality,^14^ while Levene’s test evaluated variance homogeneity.^15^ Depending on data distribution, ANOVA was employed to compare means,^16^ while the Kruskal-Wallis test served as a non-parametric alternative.^17^ Pairwise Z-tests were applied to detect significant differences in mortality rates between subgroups.^18^ A competing-risks survival analysis using the Aalen-Johansen estimator was performed to estimate the cumulative incidence of in-hospital death and discharge alive, with admission date as the time origin to ensure consistent comparisons across patients.^19^ All statistical analyses were performed in Python version 3.11.

### Ethical considerations

This study was approved by the Wayne State University IRB (IRB-23-03-5637) with a waiver of informed consent. All data were de-identified prior to analysis, and no protected health information was retained in the analytic data set.

## Results

### Descriptive analysis of total population

A total of 9,025 *Candida auris* tests were conducted between June 2023 and March 2025, with 725 positive results. Monthly testing volumes ranged from 34 tests in June 2023 to a peak of 643 tests in August 2024. The highest number of positive results (57) was also observed in August 2024, while the highest positivity rate (11.8%) occurred in December 2023. The lowest positivity rate (4.8%) was recorded in January 2025. Temporal trends in total tests, positive results, and test positivity are illustrated in Figure S1 (Supplementary). The data set did not distinguish between colonization and infection for individual tests, so these categories could not be separated in the figure.

### Demographic distribution of subpopulations

The study population was assessed based on clinical status, age, sex, race, and ethnicity. Most patients were colonized (81.97%), 11.48% were infected, and 6.56% were both. The largest age group was 61–80 years (50.41%), followed by 41–60 (29.92%), 18–40 (12.70%), and 80+ (6.97%). Males comprised 59.34% of cases. Racial distribution was predominantly African American (61.83%), followed by White (14.52%), multiple races (7.88%), and other (15.76%). Ethnicity distribution showed Not Hispanic or Latino (84.23%), Multiple Ethnicities (10.79%), and Unknown (4.97%), reflecting potential gaps in demographic reporting.

### Mortality rate analysis of subpopulations

Mortality rates varied across demographic, clinical, and glucose-related factors, reflecting complex healthcare challenges. Patients with colonized/infected status had the highest mortality (31.25%), followed by colonized-only (23%) and infected-only (21.43%) groups (as defined by Infection Control), though differences were not statistically significant (*P* = .73). Age trends showed higher mortality in middle-aged patients (41–60: 24.66%, 61–80: 25.20%), while younger (18–40: 16.13%) and oldest patients (80+: 17.65%) had lower rates, potentially due to survival bias (*P* = .68).

Among patients with *C. auris* positivity, mortality differed across racial groups (*P* = .0072), with the lowest rates observed in White patients (8.57%) and higher rates observed in Multiple-race (42.11%) and Other race (41.38%) groups. Similar patterns were observed across ethnicity categories, with the highest mortality in Multiple Ethnicity (34.62%) and Unknown (27.27%) groups, compared with Not Hispanic or Latino (21.67%) (*P* = .0072). These differences are descriptive and do not imply differential risk, as denominator data were not available for comparison.

Both glucose variability and stability were examined as complementary measures of glycemic patterning during hospitalization. Glucose variability appeared to be higher in deceased patients compared to survivors (170.58 vs 151.09 mg/dL), even though average glucose levels were similar between the two groups (136.46 vs 138.56 mg/dL; *P* = .802). However, glucose variability was not significantly associated with mortality (*P* = .209). In contrast, glucose stability, as measured by the CV, showed a significant association with mortality (*P* = .007), with the lowest mortality observed in the high stability group (9.6%), compared to the medium (30.1%) and low stability groups (25.0%).

Race, ethnicity, and glucose variability significantly influenced mortality. Among African American patients, high glucose variability corresponded to 27.3% mortality, while multiple-race patients with medium variability had the highest rate (83.3%). Other racial groups showed 60.0% mortality with high variability, while White patients had lower mortality (11.1%). Ethnicity trends followed similar patterns, with Multiple Ethnicity (40.0%) and Not Hispanic or Latino (25.4%) patients experiencing the highest mortality in the high glucose variability group. Interestingly, Unknown ethnicity patients with low glucose variability had the highest mortality (40.0%), reinforcing the complex interaction between glucose stability and demographic factors. Table 1 summarizes mortality rates across subgroups.

**Table 1.** Summary of mortality rates across demographic and clinical categories of subpopulations (June 2023 – February 2024)

Category	Subpopulation	Population size (%)^a^	Deaths (% within group; *n*)	Statistical test (*P* value)^b,c^
**Glucose variability**	Low (<84 mg/dL)	79 (33.19)	15.79% (12)	0.209
	Medium (84–190 mg/dL)	83 (34.87)	21.79% (18)	
	High (>190 mg/dL)	74 (31.93)	27.63% (20)	
**Colonized vs infected**	Colonized	198 (81.97)	23% (46)	0.73
	Colonized/Infected	16 (6.56)	31.3% (5)	
	Infected	28 (11.47)	21.4% (6)	
**Age category**	18–40	31 (12.7)	16.1% (5)	0.68
	41–60	72 (29.92)	24.7% (18)	
	61–80	122 (50.41)	25.2% (31)	
	80+	17 (6.97)	17.7% (3)	
**Sex**	Female	99 (40.66)	26.5% (26)	0.32
	Male	143 (59.34)	20.9% (30)	
**Race**	African American	148 (61.06)	20.8% (31)	0.0072***
	White	35 (14.34)	8.6% (3)	
	Multiple	19 (7.79)	42.1% (8)	
	Other	29 (12.55)	41.4% (12)	
**Ethnicity**	Multiple	26 (10.79)	34.6% (9)	0.0069***
	Not Hispanic or Latino	204 (84.23)	21.7% (44)	
	Unknown	12 (4.98)	27.3% (3)	

^a^Overall population N=242, ^b^Statistical significance is indicated by stars: p-values between 0.05 and 0.01 are marked with one star (*), p-values between 0.01 and 0.001 are marked with two stars (**), and p-values below 0.001 marked with three stars (***), ^c^ANOVA and Kruskal Wallis tests were applied. For detailed information on the methodologies and results, please refer to the Supplementary File.

### Survival analysis based on length of stay (LOS)

**Figure 1.**
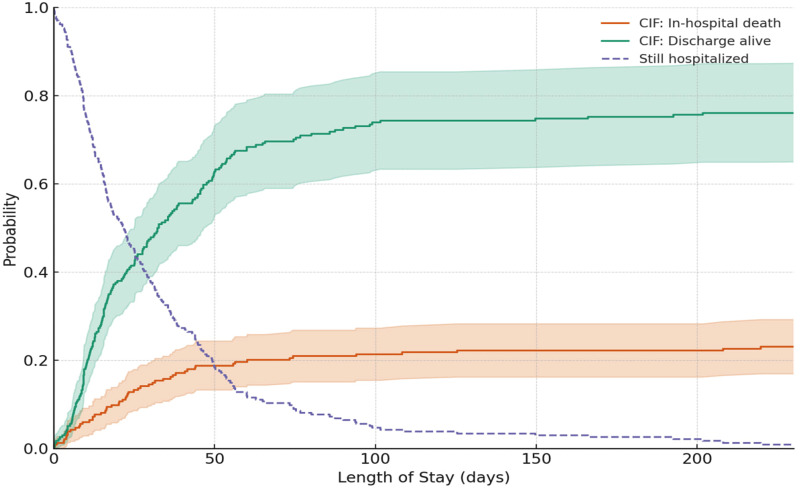
Cumulative incidence of in-hospital death, discharge alive, and probability of remaining hospitalized by length of stay (LOS), June 2023–February 2024. Curves were estimated using a competing-risks survival framework with the Aalen–Johansen estimator. Shaded regions indicate 95 % confidence intervals.

A competing-risks survival analysis was performed to estimate the probabilities of in-hospital death, discharge alive, and remaining hospitalized over time. Cumulative incidence functions were derived using the Aalen–Johansen estimator (Figure 1). Methodological details are provided in the Supplementary Materials.

### Length of stay (LOS) analysis

Patients classified as colonized/infected had an average LOS of 39.9 days (median: 27), with minimal variability. Infected-only patients exhibited the longest hospital stays (average: 67.9, median: 49), while colonized-only patients had shorter but more variable stays (30.2, median: 17), with some cases exceeding 200 days (*P* < .001).

Age-related patterns revealed that younger patients (18–40 yr) had the longest and most variable LOS (average: 56.8, median: 32.5, *P* = .032). Middle-aged groups (41–60 and 61–80 yr) showed moderate LOS (30.9 and 34.1 d, respectively), while elderly patients (80+ years) had the shortest stays (average: 18.9, median: 14).

Racial and ethnic trends showed that White patients had the longest LOS (39.0 d), African American patients had moderate stays (34.4), and Multiple-race patients had the shortest stays (20.9), but these differences were not statistically significant (*P* = .43). Ethnicity-based trends indicated longer LOS for multiple ethnicity patients (52.9 d) compared to not Hispanic or Latino patients (32.6).

Glucose variability emerged as a major determinant of LOS (*P* < .01). Patients with low glucose variability (<84 mg/dL) had the shortest stays (average: 18.75, median: 11.84). Medium glucose variability (84–190 mg/dL) was associated with extended LOS (41.3, median: 28.88), while high glucose variability (>190 mg/dL) resulted in the longest hospital stays (average: 46.14, median: 31.93).

Infection status and glucose variability emerged as the most significant determinants of LOS, with younger patients and those with high glucose variability requiring prolonged hospital stays. While age-related trends showed clear differences, sex, race, and ethnicity revealed limited statistical significance despite some variability in LOS. These findings are summarized in Table 2.

**Table 2.** Summary of length of stay (LOS) across demographic and clinical categories of subpopulations (June 2023 -February 2024)

Category	Subpopulation	Population size (%)^a^	Average length of stay per days	Median length of stay per days	Interquartile range (IQR) of length of stay	Statistical test (*P* value)^b, c^
**Glucose variability**	Low (<84 mg/dL)	79 (33.19)	18.75	11.84	17.73	0.009**
	Medium (84–190 mg/dL)	83 (34.87)	41.30	28.88	34.69	
	High (>190 mg/dL)	74 (31.93)	46.14	31.93	38.23	
**Colonized vs infected**	Colonized	198 (81.97)	30.2	17	27	0.00035***
	Colonized/Infected	16 (6.56)	39.9	27	27	
	Infected	28 (11.47)	67.9	49	45	
**Age category**	18–40	31 (12.7)	56.8	32.5	53.5	0.032*
	41–60	72 (29.92)	30.9	18	34	
	61–80	122 (50.41)	34.1	22	29.5	
	80+	17 (6.97)	18.9	14	10	
**Sex**	Female	99 (40.66)	34.4	18	34	0.87
	Male	143 (59.34)	35.5	22	33	
**Race**	African American	148 (61.06)	34.4	21	33.25	0.43
	White	35 (14.34)	39.0	27	34	
	Multiple	19 (7.79)	20.9	11	11	
	Other	29 (12.55)	45.8	23	39	
**Ethnicity**	Multiple	26 (10.79)	52.9	27	44.25	0.38
	Not Hispanic or Latino	204 (84.23)	32.6	19.5	34	
	Unknown	12 (4.98)	39.7	25	46	

^a^Overall population N=242, ^b^Statistical significance is indicated by stars: *p*-values between 0.05 and 0.01 are marked with one star (*), *p*-values between 0.01 and 0.001 are marked with two stars (**), and *p*-values below 0.001 marked with three stars (***), ^c^ANOVA and Kruskal Wallis tests were applied. For detailed information on the methodologies and results, please refer to the Supplementary File.

Since glucose variability, infection status, and age category were statistically significant factors affecting LOS, we analyzed their combined impact. Glucose instability strongly correlated with prolonged hospitalization across all age groups and infection statuses (*P* < .01). Among younger patients (18–40 yr), those with medium glucose variability had the longest LOS (91.29 d), exceeding those with low (34.35) and high variability (39.86). A similar pattern was observed in older groups (41–80 and 80+ years), where high glucose variability consistently resulted in extended LOS, compared to shorter stays in low variability patients. Infection status further amplified this effect (*P* < .001), with infected patients experiencing the longest LOS (83.67) under high glucose variability, while low variability cases had significantly shorter stays (24.58). Detailed analysis is available in Tables S1 and S2 in the supplementary material.

## Discussion

This study describes the substantial morbidity observed among patients with *C. auris* in urban Detroit, with predominance of cases among African American patients. These findings are descriptive and reflect the characteristics of patients with *C. auris* positivity within the study period; they are not intended to infer causality or comparative risk between demographic or clinical subgroups. The observed mortality rate aligns with prior national data, with infected patients experiencing high fatality rates exceeding 20%.^3^ Mortality rates were comparable between colonized and infected groups (*P* = .73), consistent with prior studies, including a large Colombian cohort reporting mixed outcomes.^20^ Patients classified as both colonized and infected had even higher mortality rates (31.3%), emphasizing the potential for colonization to progress to severe invasive disease. Nationally, mortality rates for *C. auris* infections have ranged from 30% to 60%, depending on patient comorbidities and healthcare setting.^5^ Our findings fall within this range, reinforcing the severe impact of this pathogen.

When compared to candidemia caused by other *Candida* species, *C. auris* exhibits a higher mortality rate, likely due to its multidrug resistance and late-stage diagnosis.^3,10^ Unlike *Candida albicans* infections, which have an estimated mortality rate of 15–25%, *C. auris* infections are associated with higher reported mortality, though comorbid conditions make attribution difficult.^21^ Additionally, compared to Methicillin-Resistant *Staphylococcus aureus* (MRSA) infections, which have an in-hospital mortality rate of approximately 15–20% in the U.S.,^22^ *C. auris* demonstrates a greater disease burden, further emphasizing its severity and need for aggressive management strategies.

Infected individuals had a mean LOS of 67.9 days, substantially longer than colonized patients (30.2 d). Colonized patients had a mean LOS of 30.2 days, which, while shorter than infected patients, still represents a significant burden on healthcare resources. This suggests that prolonged hospitalization among colonized patients likely reflects the burden of underlying comorbidities and infection control measures rather than colonization itself. Interestingly, younger patients (18–40 yr) experienced extended LOS (56.8), contrasting with shorter hospital stays observed in elderly patients (80+ years; 18.9). This difference likely reflects variations in clinical trajectories across age groups, without implying difference in underlying risk. Overall, the LOS among patients with *C. auris* far exceeded that reported for MRSA patients, highlighting the extensive resource utilization associated with this infection.^22^ Compared with other *Candida* species, *C. auris* has also been reported to be associated with extended hospitalizations, reflecting its clinical complexity and resource-intensive management.^23^

High glucose variability was linked to longer hospital stays, suggesting metabolic instability as a contributor to prolonged hospitalization. Variability and stability were analyzed as complementary measures of glycemic patterning, reflecting related but distinct aspects of glucose control. Prior studies on *Candida* infections show that patients with comorbidities such as diabetes and renal failure experience extended LOS.^13^ *C. auris* patients often require intensive care, further prolonging stays compared with other fungal infections. Higher glucose fluctuations correlated with both increased LOS and mortality, consistent with prior work on hyperglycemia and infection outcomes.^11,13^ Patients with high glucose variability had the highest mortality (27.63%) and prolonged stays (46.14). Although mean glucose levels were similar between survivors and non-survivors (136.46 vs 138.56 mg/dL) CV was significantly associated with mortality (*P* < .01). Because glycemic instability may reflect overall illness severity and treatment intensity, and there was no *C. auris*–negative comparator, these findings should be interpreted as associative. The impact of targeted glycemic interventions on *C. auris* outcomes warrant prospective evaluation.

The burden of *C. auris* infections reflect both individual risk factors and systemic challenges in healthcare management. The observed associations between infection status, glucose variability, and hospitalization outcomes underscore the complexity of patient care. The prolonged LOS among infected patients suggests increased healthcare resource utilization, contributing to higher treatment costs and strain on hospital systems. Furthermore, racial disparities in mortality emphasize the need for equitable healthcare strategies that strengthen infection prevention, enhance early detection for infection control, and ensure effective treatment for high-risk populations. In our study, subgroup denominators were small, and findings should be interpreted with caution and considered exploratory, underscoring the importance of infection control strategies and equitable access to effective treatment for high-risk populations.

This study has several limitations. First, its retrospective cohort design limits causal inference. Demographic distributions reflect the *C. auris*–positive population but cannot define subgroup risk because source population data were unavailable. Second, lack of infection-site details (eg, bloodstream, urinary, respiratory) limits interpretation of mortality and LOS differences. Also, classification relied on laboratory detection methods rather than clinical adjudication. Positive cultures from nonsterile sites may not indicate true infection, which may influence classification accuracy. Analyses were based on the first positive sample per patient; subsequent samples were not evaluated longitudinally. Data on confounders, including antimicrobial exposure and severity scores, were also unavailable. The single tertiary care center setting may limit generalizability. Use of glucose ranges over the entire hospitalization may partially reflect LOS and not fully capture physiologic fluctuations in glycemia. Alternative metrics such as the glucose lability index or standard deviation may provide a more nuanced assessment and warrant consideration in future studies. Measuring LOS from admission rather than detection introduces immortal time bias; analyses anchored at detection or stratified by infection status may yield further insights. Finally, while statistical approaches were applied to account for variability, small sample sizes in subgroup analyses may have reduced statistical power, and p-values were not adjusted for multiple comparisons, so findings should be interpreted with caution. Further studies with larger, more diverse populations and additional clinical variables are recommended to validate these findings.

Future research should focus on refining antifungal stewardship, developing novel therapeutics, and addressing racial disparities in outcomes.^6^ Investigations into the genetic and environmental drivers of these disparities, as well as more effective decolonization and containment strategies, are also warranted. Regional collaboration and data sharing could support better outbreak responses, as seen in strategies employed for MRSA containment.^7,25^ As *C. auris* continues to spread, integrating insights from MRSA and other fungal infections will be critical in shaping future infection control strategies.

## Conclusion

*C. auris* represents a growing healthcare challenge in urban centers,^25^ and in Detroit, African American patients account for a large proportion of reported cases.^9^ This study identifies associations between C. auris infections, increased mortality, prolonged hospitalizations, and glucose variability, highlighting the need for enhanced surveillance, infection control, and antifungal stewardship. Comparisons with MRSA and other Candida infections underscore its burden and the need for stronger prevention and treatment strategies.^10^ Future work should address drivers of disparities and evaluate interventions to improve outcomes in vulnerable populations.

## Supporting information

10.1017/ash.2025.10237.sm001 Tehaili et al. supplementary material Tehaili et al. supplementary material

## Supplementary material

The supplementary material for this article can be found at https://doi.org/10.1017/ash.2025.10237.
