# Supplementary material for: Epidemiology, length of stay, and survival outcomes of Candida auris infections in a tertiary care center in the greater detroit area
Source: Antimicrob Steward Healthc Epidemiol. 2025 Nov 27;5(1):e321. doi: 10.1017/ash.2025.10237 (PMC12722563; doi:10.1017/ash.2025.10237)
Supplement: Tehaili et al. supplementary material [file S2732494X25102374sup001.docx]

***Supplementary online content for***

**Epidemiology, Length of Stay, and Survival Outcomes of *Candida auris* Infections in a Tertiary Care Hospital in the Greater Detroit Area**

This supplementary material file has been created by the authors to give readers additional information about the research article.

A total of 9.025 *Candida auris* tests were conducted between June 2023 and March 2025, yielding 725 positive results. Monthly testing volumes ranged from 34 tests in June 2023 to a peak of 643 tests in August 2024. The highest number of positive results (57) was also observed in August 2024, while the highest positivity rate (11.8%) occurred in December 2023. In contrast, the lowest positivity rate (4.8%) was recorded in January 2025. The month-to-month distribution of total tests, positive results, and positive rates is shown in **Figure S1**.


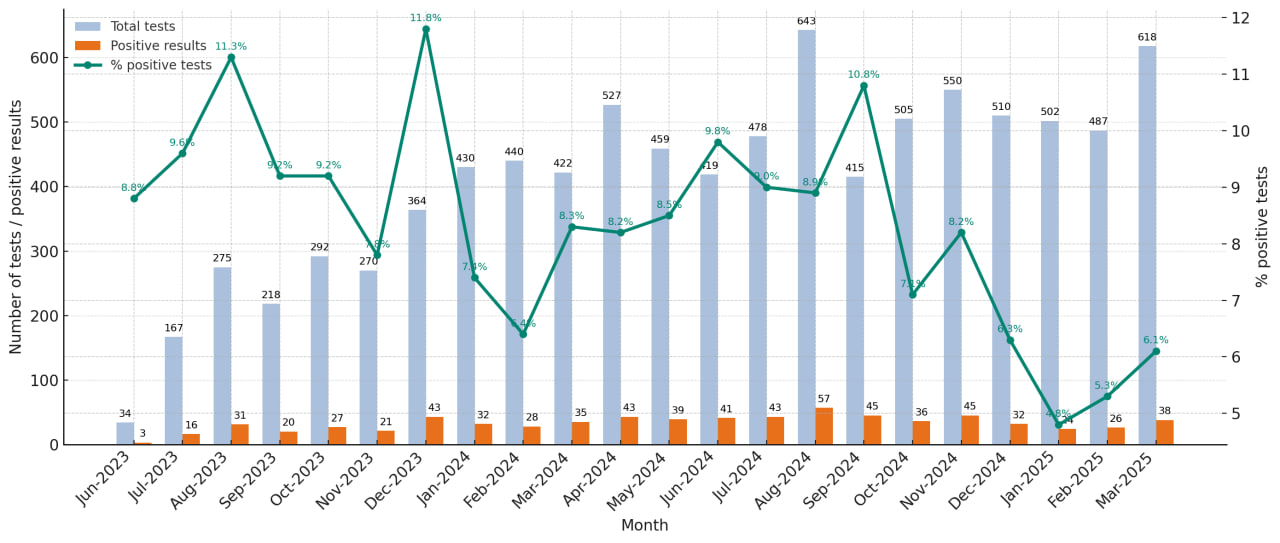


**Figure S1.** Total *Candida auris* tests (N), test positivity rate (%), and positive test cases (n) by month, June 2023–March 2025. The dataset did not include information distinguishing colonization from infection, so these categories are not separately displayed.

#### Length of Stay (LOS) Based on Glucose Variability, Age Category:

Since glucose variability, infection status, and age category significantly impact LOS (p < 0.01), we performed a more detailed analysis by incorporating both glucose variability (mg/dL) and glucose Coefficient of Variation (CV%), a widely used metric for assessing glucose fluctuations. Higher glucose variability was consistently associated with longer hospitalization, and this pattern remained evident across all stratifications, reinforcing the importance of glycemic control in Candida auris patients.

Across age groups, glucose variability strongly influenced LOS, with younger patients (18–40 years) experiencing the most pronounced effects. Patients in this group with moderate glucose variability had the longest LOS (91.29 days), compared to 34.35 days in the low variability group and 39.86 days in the high variability group (Table S1). This pattern was also observed in the 41–60 age group, where high glucose variability was linked to an extended LOS of 55.09 days, while low glucose variability patients had significantly shorter hospitalizations (13.81 days). The 61–80 age category followed a similar trend, with high glucose variability patients averaging 44.87 days compared to 18.76 days in the low variability group. Among elderly patients (80+ years), those with high glucose variability had an average LOS of 30.14 days, while low variability patients had the shortest stays (9.50 days). When analyzing glucose variability using CV%, a similar pattern emerged, where moderate glucose fluctuations led to the longest LOS across all age groups. For example, in patients aged 18–40, those with moderate CV% had an average LOS of 80.95 days, higher than both high CV% (35.58 days) and low CV% (38.44 days), suggesting that sustained moderate glucose instability is particularly detrimental. This trend was consistent across all age groups, with moderate glucose fluctuations correlating with prolonged hospitalization.

Stratifying LOS by infection status (colonized, colonized/infected, and infected) further confirmed a significant relationship between glucose variability and LOS (p < 0.001). Infected patients with high glucose variability had the longest LOS (83.67 days), whereas those with low variability had significantly shorter stays (24.58 days). A similar trend was seen in colonized/infected patients, where higher glucose variability was associated with an extended LOS of 38.90 days compared to 29.90 days in those with low glucose variability. Among colonized-only patients, high glucose variability cases exhibited an average LOS of 38.77 days, while low variability cases had a significantly shorter LOS of 17.88 days. Further analysis using glucose CV% reinforced these findings. Among infected patients, moderate CV% cases exhibited the longest LOS (71.52 days), followed by high CV% (60.29 days) and low CV% (37.25 days). Interestingly, in colonized/infected patients, moderate glucose variability remained a key determinant, with a LOS of 56.59 days, while high CV% patients had a significantly lower LOS (15.99 days) (Table S2). This inverse trend in high CV% cases suggests that extreme glucose fluctuations may not have the same effect as moderate instability in certain patient subgroups, a finding that warrants further investigation. Among colonized patients, moderate CV% was again associated with prolonged hospitalization (37.94 days), while both high and low CV% groups had similar LOS values (27.19 and 27.49 days, respectively). These findings, summarized in Table S1.

**Table S1**: Summary of Length of Stay (LOS) Based on Glucose Variability and Glucose Coefficient of Variation (CV%) Across Age Categories.

| **Category** | **Subcategory** | **Glucose Variability** | **Number of Cases** | **Average Length of Stay per Days** | **Median Length of Stay per Days** | **Interquartile Range** (**IQR) of Length of Stay** | **Statistical Test (p-value)** |
| --- | --- | --- | --- | --- | --- | --- | --- |
| **Age** | 18-40 | High | 9 | 39 | 44.74 | 47.26 | 0.041 |
|  |  | Medium | 9 | 91.29 | 38.32 | 41.08 |  |
|  |  | Low | 10 | 34.35 | 10.76 | 16.98 |  |
|  | 41-60 | High | 20 | 55.09 | 43.49 | 37.06 | 0.002 |
|  |  | Medium | 27 | 32.92 | 25.21 | 32.08 |  |
|  |  | Low | 24 | 13.81 | 10.39 | 10.04 |  |
|  | 61-80 | High | 33 | 44.86 | 26.25 | 27.78 | 0.015 |
|  |  | Medium | 43 | 38.11 | 27.64 | 35.9 |  |
|  |  | Low | 41 | 18.75 | 14.71 | 21.3 |  |
|  | 80+ | High | 5 | 30.14 | 15.13 | 42.8 | 0.089 |
|  |  | Medium | 4 | 19.73 | 18.59 | 10.67 |  |
|  |  | Low | 4 | 9.5 | 10.01 | 8.35 |  |
| **Category** | **Subcategory** | Glucose Coefficient of Variation (CV%) | **Number of Cases** | **Average Length of Stay per Days** | **Median Length of Stay per Days** | **Interquartile Range** (**IQR) of Length of Stay** | **Statistical Test (p-value)** |
| **Age** | 18-40 | High | 7 | 35.58 | 31.94 | 43.23 | 0.038 |
|  |  | Moderate | 11 | 80.95 | 38.32 | 44.96 |  |
|  |  | Low | 10 | 38.44 | 17.68 | 32.06 |  |
|  | 41-60 | High | 14 | 46.07 | 40.26 | 45.49 | 0.045 |
|  |  | Moderate | 37 | 33.07 | 22.13 | 34.62 |  |
|  |  | Low | 17 | 26.59 | 16.99 | 22.56 |  |
|  | 61-80 | High | 24 | 21.71 | 17.19 | 14.87 | 0.022 |
|  |  | Moderate | 52 | 44.95 | 27.59 | 38.56 |  |
|  |  | Low | 37 | 27.63 | 25.28 | 26.69 |  |
|  | 80+ | High | 4 | 23.59 | 13.89 | 12.91 | 0.057 |
|  |  | Moderate | 4 | 28.43 | 24.18 | 24.69 |  |
|  |  | Low | 4 | 14.41 | 14.59 | 5.58 |  |

**Table S2**: Summary of Length of Stay (LOS) Based on Glucose Variability and Glucose Coefficient of Variation (CV%) Across Infectious Categories.

| **Category** | **Subcategory** | **Glucose Variability** | **Number of Cases** | **Average Length of Stay per Days** | **Median Length of Stay per Days** | **Interquartile Range** (**IQR) of Length of Stay** | **Statistical Test (p-value)** |
| --- | --- | --- | --- | --- | --- | --- | --- |
| **Colonized vs Infected** | Colonized | High | 51 | 38.77 | 29.02 | 34.63 | 0.00013 |
|  |  | Medium | 65 | 38.85 | 25.21 | 25.68 |  |
|  |  | Low | 71 | 17.88 | 11.14 | 15.87 |  |
|  | Colonized/Infected | High | 5 | 38.90 | 26.25 | 5 | 0.0452 |
|  |  | Medium | 7 | 46.07 | 31.46 | 31.8 |  |
|  |  | Low | 3 | 29.90 | 32.62 | 21.67 |  |
|  | Infected | High | 11 | 83.66 | 54.9 | 41.85 | 0.0078 |
|  |  | Medium | 11 | 52.78 | 52.04 | 22.89 |  |
|  |  | Low | 5 | 24.58 | 18.36 | 20.68 |  |
| **Category** | **Subcategory** | Glucose Coefficient of Variation (CV%) | **Number of Cases** | **Average Length of Stay per Days** | **Median Length of Stay per Days** | **Interquartile Range** (**IQR) of Length of Stay** | **Statistical Test (p-value)** |
| **Colonized vs Infected** | Colonized | High | 40 | 27.49 | 16.59 | 30.21 | 0.00025 |
|  |  | Moderate | 82 | 55.83 | 22.38 | 27.86 |  |
|  |  | Low | 57 | 25.82 | 19.21 | 24.87 |  |
|  | Colonized/Infected | High | 3 | 6.95 | 16.68 | 6.98 | 0.0389 |
|  |  | Moderate | 8 | 52.09 | 33.84 | 38.44 |  |
|  |  | Low | 4 | 19.03 | 24.52 | 30.14 |  |
|  | Infected | High | 6 | 66.97 | 44.44 | 22.49 | 0.0043 |
|  |  | Moderate | 14 | 62.39 | 55.63 | 28.78 |  |
|  |  | Low | 7 | 25.17 | 35.55 | 34.22 |  |

#### Survival Analysis Based on Length of Stay (LOS)

A competing-risks survival framework was used to estimate the probabilities of in-hospital death, discharge alive, and remaining hospitalized over time. This approach accounts for the non-independence between discharge and death by treating them as mutually exclusive outcomes. Cumulative incidence functions (CIFs) were derived using the Aalen–Johansen estimator^1,2^, with 95% confidence bands computed using a Greenwood-type variance approximation^3^.


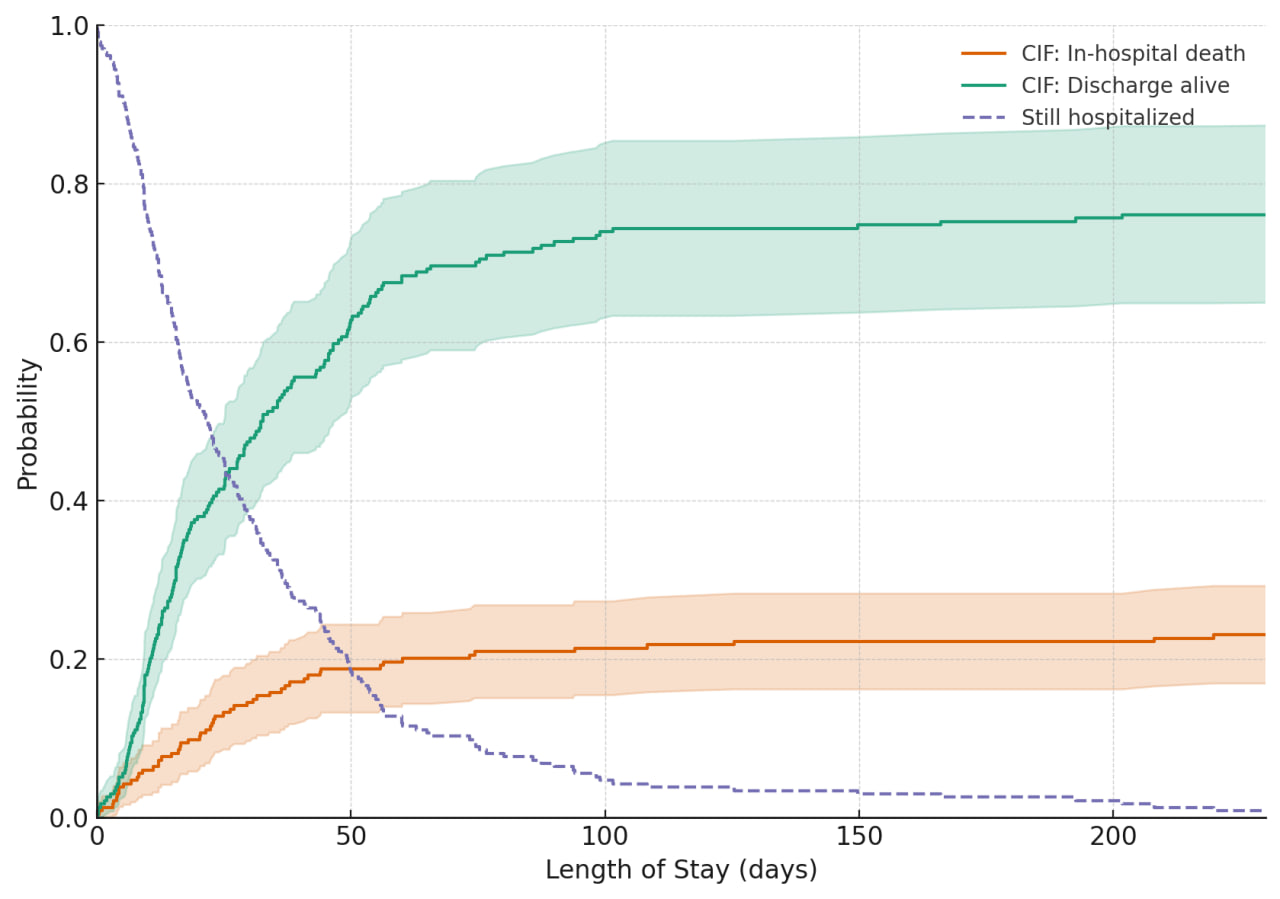


**Figure 1.** Cumulative incidence of in-hospital death, discharge alive, and probability of remaining hospitalized based on length of stay (LOS), June 2023–February 2024.

The Aalen–Johansen estimator was applied to derive CIFs for each outcome type. This nonparametric estimator models patient transitions between multiple states and updates the probability of remaining in the initial state as events occur over time. Specifically, for each event type *k*, the cumulative incidence function is defined as:

$$\hat{F}_{k}(t)=\sum_{t_{i}\leq t} \hat{S}\left( t_{i-1} \right)\frac{d_{ik}}{n_{i}}$$

where

- $\hat{F}_{k}(t)$ is the cumulative probability of event type *k* (e.g., death or discharge) by time *t*,
- $\hat{S}(t_{i-1})$ represents the estimated probability of remaining hospitalized immediately before time $t_{i}$,
- $d_{ik}$is the number of observed events of type *k* at time titi​, and
- $n_{i}$ denotes the number of patients still at risk at time $t_{i}$​.

The probability of remaining in the initial state is updated recursively as:

$$\hat{S}(t_{j})=\hat{S}(t_{j-1})(1-\frac{\sum_{k} d_{jk}}{n_{j}})$$

To quantify uncertainty, 95% confidence bands were constructed using a Greenwood-type variance approximation, which provides a nonparametric estimate of the variability in the CIFs. This allows the calculation of time-dependent probabilities for each outcome while reflecting increased uncertainty in later hospitalization days when fewer patients remain under observation.

In this analysis, event type 1 corresponded to in-hospital death, event type 2 to discharge alive, and individuals who experienced neither event by the end of follow-up were considered right-censored (still hospitalized). The estimated CIFs and confidence intervals are presented in **Figure 1**, demonstrating the evolution of these probabilities over the study period.

### Supplementary References:

1. Mansournia MA, Nazemipour M, Etminan M. A practical guide to handling competing events in etiologic time-to-event studies. *Glob Epidemiol.* 2022;4:100080.
2. Putter H, Fiocco M, Geskus RB. Tutorial in biostatistics: competing risks and multi‐state models. *Stat Med.*2007;26(11):2389–2430.
3. Allignol A, Schumacher M, Beyersmann J. Empirical transition matrix of multi-state models: the *etm* package. *J Stat Softw.* 2011;38(4):1–15.
